# Extraction of the Microstructure of Wool Fabrics Based on Structure Tensor

**DOI:** 10.3390/s23156813

**Published:** 2023-07-31

**Authors:** Jiani Zhu, Youwei Ma, Guoqing Ding, Manhua Liu, Xin Chen

**Affiliations:** 1 School of Electronic Information and Electrical Engineering, Shanghai Jiao Tong University, Shanghai 200240, China; 2 Shanghai Aerospace Control Technology Institute, Shanghai 201109, China

**Keywords:** fabrics, CT analysis, 3D reconstruction, microsturcture parameters

## Abstract

The trends of “fashionalization”, “personalization” and “customization” of wool fabrics have prompted the textile industry to change the original processing design based on the experience of engineers and trial production. In order to adapt to the promotion of intelligent production, the microstructure of wool fabrics is introduced into the finishing process. This article presents an automated method to extract the microstructure from the micro-CT data of woven wool fabrics. Firstly, image processing was performed on the 3D micro-CT images of the fabric. The raw grayscale data were converted into eigenvectors of the structure tensor to segment the individual yarns. These data were then used to calculate the three parameters of diameter, spacing and the path of the center points of the yarn for the microstructure. The experimental results showed that the proposed method was quite accurate and robust on woven single-ply tweed fabrics.

## 1. Introduction

Wool has good inherent properties and appearance characteristics. It has always been a popular and high-grade fabric. With the improvement of living standards, the demand for wool fabric is also more diversified and has begun to pursue “fashionalization”, “personalization” and “customization” [1,2].

The feeling that a piece of fabric brings to the eye, touch and other senses is often described as the style. The textile industry defines hundreds of styles for different types of materials for various purposes, which are intricately and intrinsically linked to each other and together determine the fabric’s quality.

Styles are usually evaluated on the basis of personal experience, supplemented by a few physical quantities measured by instruments. The results are generally highly subjective and vary individually. In addition, the expression of the customer’s needs is often colloquial and comparative in nature. The producer’s understanding of the customer’s requirements is prone to bias. Agreement between the parties may require several exchanges, which will undoubtedly reduce productivity and the customer’s experience.

Customization means processing products with different requirements in small lots and multiple batches, and with high efficiency. In the past, the design process of mass production usually used the process of trying to produce a small number of samples, comparing the differences between the samples and the requirements, adjusting the process, trying to produce samples again and comparing them again. If this process is used in customization, it will undoubtedly consume excessive time and material costs and deter potential customers [3].

**Figure 1:**
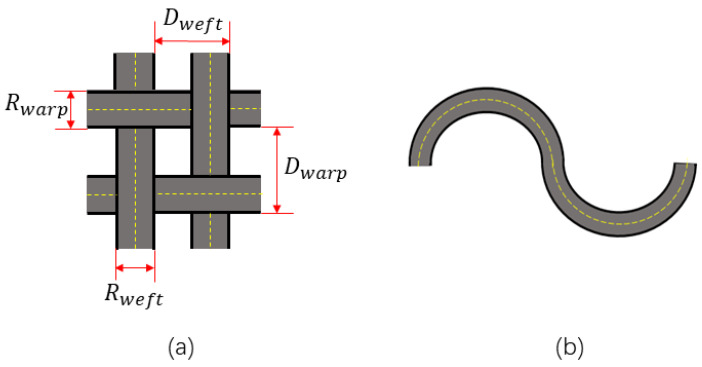
Microstructure of woven fabrics: (**a**) top view; (**b**) side view. The path of the center points is indicated by the yellow dashed line.

In the face of the problems above, textile companies are seeking to quantify the properties of wool fabrics in order to establish a mathematical link between the processing process and the finished style, and to further realize the automated design of the processing process. Based on the theory that the microstructure of a fabric determines its macroscopic properties [4,5,6,7,8,9], it is feasible to introduce the microstructure instead of traditional quality control methods in the selection of raw materials, the design process and the production processes of fabric processing [10]. There are three main geometrically described parameters (shown in Figure 1) that characterize the microstructure: the yarn’s properties (e.g., yarn diameter R), the yarn’s spacing D and the topology, expressed in terms of the path of the center points [11].

Manual measurement is a straightforward method used to obtain the microstructure of wool fabrics, which is time-consuming and laborious. Traditional measurement tools (e.g., calipers) are applied to individual yarns disassembled from the fabric, which breaks the mutual constraint between the yarns and causes errors in the results of measurement. 

With the widening application of computer vision in the textile industry [12,13,14], it is more convenient to process the high-definition top view images of fabric for feature extraction. Luan et al. [15] used the polarization properties of yarns for accurate segmentation of the warps and wefts, overcoming the requirement of other methods regarding the number of colors contained in the textile and allowing the processing of fabrics consisting of only two colors. Fang et al. [16] obtained the position of the yarn from the projected image of the fabric and the texture from the reflected image, which could effectively handle fabrics where the warps and wefts are in different colors. Xiang et al. [17] improved the luminance projection method to locate yarns and knots, which is effective for extracting the features of yarn-dyed fabric. A calibration method for cell phone photos [18] was proposed to reduce the requirement for instruments and to achieve real-time measurements.

More studies have been based on neutral networks to label the region or trajectory of yarns in an image. The extraction network of a texture’s structure proposed by Yuan et al. [19] extracted periodic texture information from fabric images to accomplish yarn segmentation. Dai et al. [20] designed a network consisting of a dilated feature network and a feature alignment module to detect each segment of the yarn based on rotating object detection. Meng et al. [21] improved the learning of yarn features by detecting yarns and floats in multitask learning.

However, the methods mentioned above only analyze the fabric’s structure at the two-dimensional level and cannot obtain third-dimensional information such as the interleafing relationship between the yarns.

Micro-CT images can capture the internal three-dimensional structure of a material with high precision in a non-destructive manner [22] and can effectively obtain the full information of the fabric’s microstructure [23]. On this basis, Shinohara et al. [24] designed a correlation function between a cylindrical model of an ideal yarn and its real voxel. The real voxel was matched to the model by maximizing the value of the function to obtain information on the position of each yarn. As a method applicable to fabrics of various tissues, it faces the problem that the computation requires iterations and priori knowledge.

More methods tend to slice the 3D image pixel by pixel along a certain coordinate axis rather than processing the stereo information directly. The slices are processed individually and then stitched back to the original structure. By slicing in the direction parallel to the fabric’s plane, excellent networks in the field of image segmentation such as U-Net [25,26] or DCNN [27] were used to label the warp and weft segments on the slices. 

It is more common to slice the image in a direction perpendicular to the plane of fabric. Pidou-Brion et al. [28] proposed a yarn segmentation method with deformable meshes to fit the fault volume of the yarn to achieve the modeling of the internal structure of composites at the mesoscale.

The most common approach is still the semantic segmentation of slices using deep learning. The segmentation network proposed by Guo et al. [29] combined coarse-to-fine segmentation and region-wise segmentation to achieve accurate segmentation of the yarn’s cross-sections. Song et al. [30] used Leaky ReLu as the activation function of U-Net to improve the robustness and efficiency of the network. The identification of binder yarns in composites was enhanced by DCNN by Ali et al. [31].

Training neural networks usually requires a large number of learning samples. There is often a lack of available public datasets in the textile field. In order to reduce expensive manual annotation, training samples can be obtained by generating pseudo-images [32] or pseudo-labels [33] when different slices are said to have similar cross-sectional structures. Zheng et al. [34] proposed a new data augmentation algorithm to generate realistic artificial datasets. Applying the transfer learning strategy [35] by leveraging networks trained on other samples can also effectively reduce the need for data when learning new samples.

**Figure 2:**
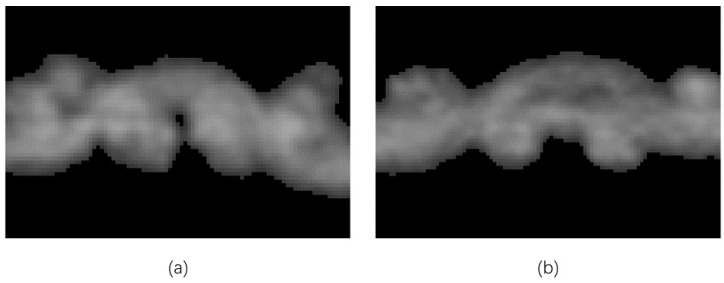
Cross-sections of wool fabric: (**a**) the case where the warps are perpendicular to the slicing plane; (**b**) the case where the wefts are perpendicular to the slicing plane.

The objects of the methods above are mainly composite materials made of carbon fibers. The cross-section has a regular shape with clear edges and good repeatability on different slices. In contrast, wool yarn is a soft, easily deformable substance. After the fabric is made, the warps and wefts are usually tightly adhered. The cross-section (shown in Figure 2) is mostly blurred at the edges and has no fixed shape. This severely increases the difficulty of creating annotations for datasets, making deep learning unfeasible.

Another practice is to identify the cross-sections of yarns perpendicular to the plane of the slices by template matching and then to reconstruct the stereoscopic shape of the yarn [36,37,38]. The template is usually a circle or an ellipse that approximates the shape of the yarn’s cross-section [39], or a manually calibrated initial cross-section of the yarn [36].

The template methods are usually used to split yarns or fibers that are aligned in the same direction. The slice of fabric usually contains both yarns parallel to the plane of the slice and yarns perpendicular to it. The former are usually larger in area and have higher and more uniform gray values, making it easier to pass the template’s matching conditions than the latter. This ultimately leads to incorrect yarn profiles and an illogical path of the center points.

In this article, a yarn segmentation method based on the structure tensor is proposed for the unique characteristics of wool fabrics. Yarn segmentation is performed by replacing the gray values with eigenvectors of the structure tensor as the feature analyzed by the template tracking method to extract the three parameters of the yarn, namely the diameter, spacing and the center points’ path, which characterize the microstructure. 

The remainder of this article is structured as follows. The experimental samples, the data acquisition method and the proposed algorithm are described in detail in Section 2. Section 3 contains the experimental results, and Section 4 presents the conclusions.

## 2. Materials and Methods

The algorithm proposed for extracting wool fabric’s microstructural features from micro-CT images is divided into two steps: yarn segmentation from micro-CT images and extraction of the feature parameters based on the segmentation results. The acquisition and preprocessing of the raw data are explained in Section 2.1. The next two sections interpret, in detail, the two parts of the yarn segmentation algorithm: feature transformation based on the structure tensor (Section 2.2) and the template selection algorithm based on the new features (Section 2.3). Section 2.4 illustrates the post-processing of yarn segmentation. The extraction of the feature parameters is accounted for in Section 2.5.

### 2.1. Materials and Preprocessing

**Figure 3:**
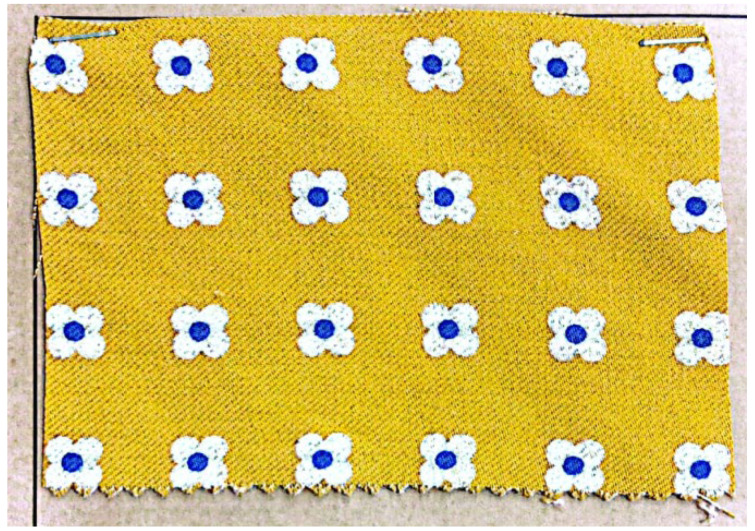
One of the samples used in the experiment.

The samples used were woven single-ply tweed fabrics. Figure 3 shows a physical image of one of the samples, with the basic information shown in Table 1. The basic information comes from the design sheet of the sample, except for the density of yarns. The density of yarns, meaning the number of yarns contained in a unit of length, was obtained by manually counting the number of yarns within 2 cm and then scaling up. The warp of the sample was made of twisted double-stranded yarn and the weft was single-stranded yarn, which are the two most common yarn styles. The ROI of this study, i.e., the minimum repeat unit of the sample’s texture, was four warps by four wefts.

Images of the samples were acquired by a micro-CT scanner, the details of which are recorded in Appendix A. The sample was fixed vertically on the sample table by a rectangular carbon fiber plate with inner size of 25 × 20 mm and was rotated to acquire the information on the two-dimensional projection inside the sample. The operating parameters (recorded in Table 2) were set according to the technical manual of the instrument and previous experiments in our laboratory.

**Figure 4:**
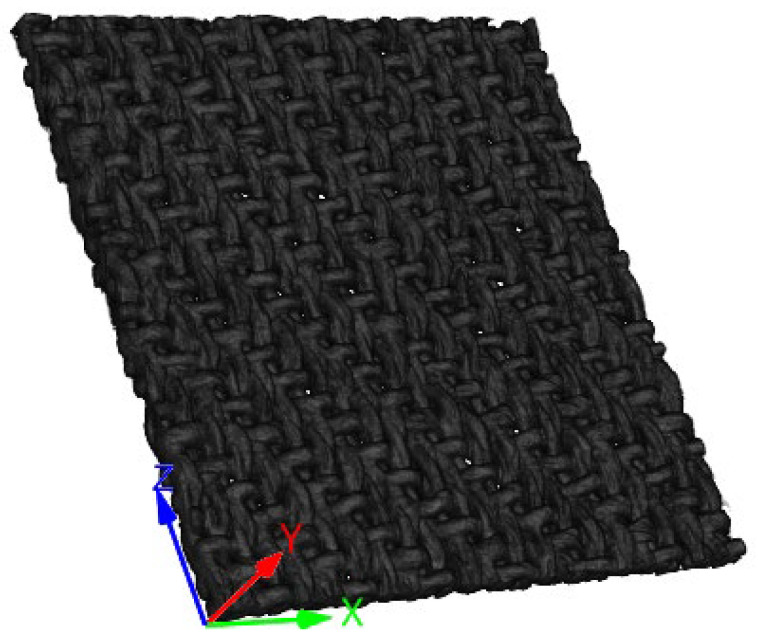
A 3D volume image of the sample.

The 2D projection images of the samples were reconstructed into 3D volume images and corrected for annular artifacts by the reconstruction software NRecon. The background corresponding to air was introduced into the reconstruction process with noise. The OTSU method [40] was used to calculate a global threshold for the image to split the foreground and background. The pixels in the background were set to 0. The sample had a natural inhomogeneity, and the reconstruction process also introduced noise in the foreground. A Gaussian filter was applied in order to smooth it while preserving the gray distribution of the image as much as possible. The image was then normalized to (0–255). The result is shown in Figure 4. For the coherence of the text, the detailed results of each step of preprocessing are recorded in Appendix B. In the following steps, the *X*, *Y* and *Z* axes of the 3D volume image were aligned with the directions of the weft, thickness and warp, respectively.

The fine hairiness on the yarn’s surface and lint with low grayscale values introduced errors in estimating the yarn’s diameter and fitting the path of the center points. Before segmenting the yarns, they needed to be removed. Only the tightly structured central part of the yarn should be retained. Mathematical morphology is a method of image analysis that uses structural elements to identify and extract shapes in an image that resemble structural elements [41], consisting of four basic operations: erosion, expansion, open operation and closed operation. Open operation first erodes and then expands the image, which can effectively remove small objects that do not contain structural elements other than the main object. Open operation was performed on the 3D volume image with a spherical structural element. The connected domain with the highest number of non-zero pixels was retained.

### 2.2. Structure Tensor

The gray value of volume image indicates the capacity to absorb X-rays. The higher the gray value, the higher the absorption capacity. For wool fabrics, the X-ray absorption capacity is positively correlated with density. The density inside the yarn decreases from the center to the periphery in the axial direction, and remains similar in the radial direction, showing a clear anisotropy.

Anisotropy can be characterized by a structure tensor. The eigenvector of the structure tensor corresponding to its smallest eigenvalue is parallel to the direction of the smallest change in the grayscale value [42]. That is, the eigenvalues of the pixels within the yarn are theoretically parallel to the axial direction of the yarn. The structure tensor is calculated by the following equation [43]

(1) $$S\left(p\right)=\left[\begin{array}{ccc} {\langle \frac{\partial I}{\partial x}\frac{\partial I}{\partial x}\rangle }_{N} & {\langle \frac{\partial I}{\partial x}\frac{\partial I}{\partial y}\rangle }_{N} & {\langle \frac{\partial I}{\partial x}\frac{\partial I}{\partial z}\rangle }_{N}\\ {\langle \frac{\partial I}{\partial y}\frac{\partial I}{\partial x}\rangle }_{N} & {\langle \frac{\partial I}{\partial y}\frac{\partial I}{\partial y}\rangle }_{N} & {\langle \frac{\partial I}{\partial y}\frac{\partial I}{\partial z}\rangle }_{N}\\ {\langle \frac{\partial I}{\partial z}\frac{\partial I}{\partial x}\rangle }_{N} & {\langle \frac{\partial I}{\partial z}\frac{\partial I}{\partial y}\rangle }_{N} & {\langle \frac{\partial I}{\partial z}\frac{\partial I}{\partial z}\rangle }_{N} \end{array}\right]$$

where I denotes the grayscale image, and ${\langle \ldots \rangle }_{N}$ denotes the mean value of all pixel points within the cubic region centered on $p=(x_{p},y_{p},z_{p})$, which is the pixel point to be calculated with a side length of 2ω+1:

(2) $$\left\{x,y,z|\left(\left|x-x_{p}\right|\leq \omega \right),\left(\left|y-y_{p}\right|\leq \omega \right),\left(\left|z-z_{p}\right|\leq \omega \right)\right\}$$

The derivatives are calculated using the five-point differential formula, which is shown below for the *x*-axis direction [43], and the same formula is used for the *y*- and *z*-axis directions.

(3) $$\frac{\partial I}{\partial x}=\frac{I\left(x-2,y,z\right)-8I\left(x-1,y,z\right)+8I\left(x+1,y,z\right)-I(x+2,y,z)}{12}$$

The eigenvector $E=(e_{1},e_{2},e_{3})$ corresponding to the smallest eigenvalue is projected to a plane perpendicular to the radial axis, and the absolute value of the orientation ϕ is then determined. The warps’ eigenvector is projected to the Y–X plane. The orientation is determined as follows:

(4) $$\phi =abs(atan2(e_{2},e_{1}))$$

**Figure 5:**
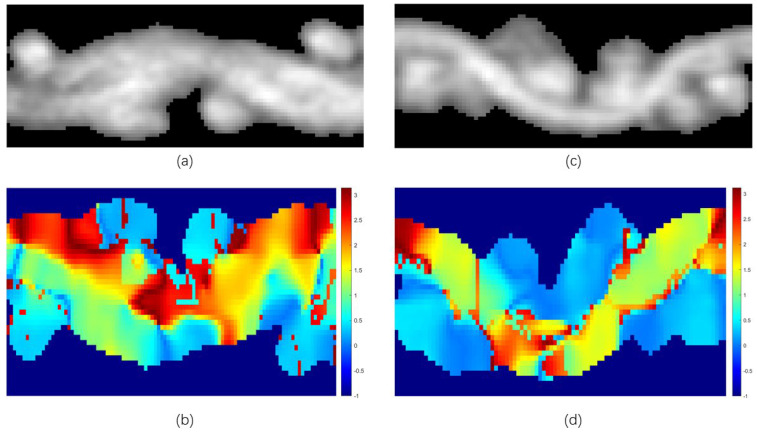
Orientation maps of the yarns. (**a**) Original grayscale image of a slice in the *Y*–*Z* plane. (**b**) Orientation map projected in the *Y*–*Z* plane. (**c**) Original grayscale image of a slice in the *Y*–*X* plane; (**d**) Orientation map projected in the *Y*–*X* plane. For display purposes, the orientation corresponding to the zero-valued pixels in the original image have been modified to −1.

The equation is then derived by projecting the eigenvector of the wefts to the *Y*–*Z* plane. In Figure 5, a diagram of the orientation of the warps and wefts is shown. The orientation of the yarns that are radially perpendicular to the slice plane is near 0°, and those parallel to it are near 90°. In other words, the area where the sum of the orientation is the smallest is the cross-section of the yarn.

### 2.3. Segmentation by Template

Before comparing the value of the orientation as a new feature of the volume image, a preliminary prediction of the yarn’s dimensions is needed to determine the range to be included in the sum, i.e., an elliptical template needs to be created for each yarn. 

The cross-section area varies less perpendicular to the radial direction and varies more parallel to the radial direction. The slice corresponding to the local minimum of the total number of non-zero pixels is between two yarns parallel to the slice plane and can be used to coarsely divide the yarns. This slice contains the least area of yarns of other directions and interferes the least with the accuracy of the cross-section’s annotation. This slice is processed in the same way to discriminate the cross-sections of the yarns.

**Figure 6:**
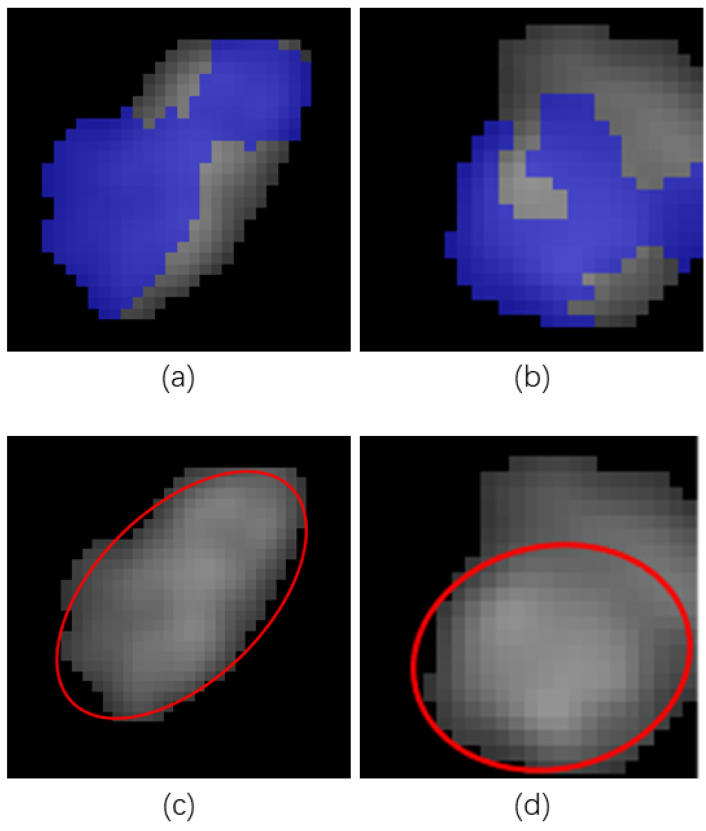
The results of labeling the yarn’s cross-section and the fitted elliptical template. (**a**) Results of labeling a warp’s cross-section; (**b**) results of labeling a weft’s cross-section (**c**) elliptical template of the warp’s cross-section; (**d**) elliptical template of the weft’s cross-section. The labelled areas are illustrated by blue and the corresponding elliptical templates are shown in red.

In most cases, the cross-sections of the yarns selected by the process above are separately connected components, which are occasionally connected to a yarn in another direction. The cross-section is labeled using the region growth algorithm [44] with the seed set to the pixel with the highest grayscale value. The center of the yarn is denser than the edges and absorbs more X-rays, and the pixel with the highest gray value is usually in the center of the cross-section. The growth condition is

(5) $$\left|\phi_{i}-\phi_{mean}\right|<1$$

where $\phi_{i}$ denotes the orientation of the pixel point to be judged and $\phi_{mean}$ denotes the average orientation angle of the selected pixel points. Figure 6a,b shows the results of labeling a set of warps and wefts.

**Figure 7:**
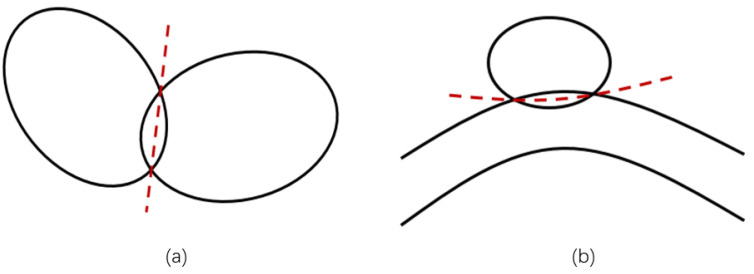
Schematic diagram of the post-processing methods: (**a**) processing of the overlap between adjacent warps or wefts; (**b**) processing of the overlap in the area where the warp and weft threads intersect. The solid black line indicates the outline of the yarn’s cross-section and the red dotted line indicates the division line of the overlap.

The parameters of the elliptical template generated by the cross-section are calculated by the geometric moment and central moment [45]. Considering that the shape of the cross-section is the main object of interest, the image is binarized to eliminate the effect of grayscale. That is, all non-zero pixels in the cross-section are assigned a value of 1, representing the foreground. The equations for the horizontal and vertical coordinates (x,y) of the central point, the radius of the major axis a and of the minor axis b, and the inclination angle θ of the major axis are as shown below [46]

(6) $$x=\frac{m_{10}}{m_{00}},y=\frac{m_{01}}{m_{00}}$$

(7) $$a=\sqrt{\frac{2\left(\mu_{20}+\mu_{02}+\sqrt{{\left(\mu_{20}-\mu_{02}\right)}^{2}+4{\mu_{11}}^{2}}\right)}{\mu_{00}}}$$

(8) $$b=\sqrt{\frac{2(\mu_{20}+\mu_{02}-\sqrt{{\left(\mu_{20}-\mu_{02}\right)}^{2}+4{\mu_{11}}^{2}})}{\mu_{00}}}$$

(9) $$\theta =\frac{1}{2}arctan(\frac{2\mu_{11}}{\mu_{20}-\mu_{02}})$$

where $m_{pq}$ and $\mu_{pq}$ denote the (p + q)-order geometric moment and the central moment, respectively; the mass center used in the calculation of the central moment is the same as the center point of the ellipse. Figure 6c and Figure 7d show the elliptical templates built on the basis of the labeling results.

The dimensions of the cross-sections (the radius of major axis a and of minor axis b) in the adjacent slices are almost constant. The variations are mainly in the center point coordinates $\left(\bar{x},\bar{y}\right)$ for both warps and wefts, and in the inclination angle θ for the double-stranded yarn. The vertical coordinates of the center point vary much more than the horizontal coordinates in the small ROI, and the line of the horizontal coordinates in all slices can be approximated as a straight line.

The position of the cross-section is traced to both sides, starting from the initial slice where the template is created. The vertical coordinate ${\bar{y}}_{i}$ of the center point of the cross-section in the current slice i is shifted in steps of 1 pixel in the range $\left[y_{i-1}-5,y_{i-1}+5\right]$, and the horizontal coordinate ${\bar{x}}_{i}$ is shifted in steps of 0.2 pixels in $\left[x_{1}-2,x_{1}-2\right]$. ${\bar{y}}_{i-1}$ denotes the vertical coordinate of the center point of the cross-section in the previous slice i−1, and ${\bar{x}}_{1}$ denotes the initial value of horizontal coordinate. The inclination angle θ is varied in steps of 5° within [0°, 180°].

If the horizontal coordinate is also shifted from the value on the previous slice, it may accumulate errors to the extent that the result of labeling shifts to the adjacent yarn, so the range of the horizontal coordinate is limited by using the initial value as the base point. 

Before calculating the sum of orientation angles of all pixels within the template, it is necessary to first use RLOESS smoothing [47] on the orientation to remove outliers. The orientation of zero-valued pixels is set to 90°, which means that yarns parallel to the slice plane are transformed into the background.

The smaller the sum of all pixel orientation angles, the more pixels within the template that belong to yarns perpendicular to the slice’s plane. The area where the minimum value of the sum changes the least compared with the previous slice is chosen as the cross-section of the yarn in that slice. By connecting all slices in series, the basic shape of the yarn can be obtained.

### 2.4. Post-Processing

The yarns extracted after the method above still have the following problems: (1) overlaps between adjacent warps or adjacent wefts, (2) overlaps in the region of the intersection of warps and wefts, and (3) residual regions. The following treatments are applied to optimize them, respectively.

1. The overlap between adjacent warps or adjacent wefts is processed in slicing order. The line connecting the highest point and the lowest point of the overlapping region on the slice is used as the new boundary of these two yarns (shown in Figure 7a).
2. The overlap of the intersecting regions of the warps and wefts are sliced by the *X*-axis or the *Z*-axis, which, in the slice, is divided equally by a column (shown in Figure 7b).
3. Pixels without an attribution are given the label of the yarn with the smallest Euclidean distance from it (and a distance less than the threshold).

### 2.5. Feature Extraction

The characteristics of the microstructure of the wool fabric were obtained by analyzing the cross-sections of the split yarns after slicing along the coordinate axis parallel to their axes.

1. Path of the center points: Usually, the path of the center points is fitted by B splines [48]. However, after calculation of the center points of the cross-section on each slice by Equation (6), no further interpolation was required. The discrete data of the center points were noisy. The approximate path of the center points was smoothed by polynomial fitting. The horizontal coordinate of the center point was approximated as a straight line within the ROI, so the relationship between $\bar{x}$ and slice coordinates could be fitted as a first-order polynomial. The path of the vertical coordinate was more complex and approximated a wavy line. The relationship between $\bar{y}$ and the slice’s coordinates was fitted as 6-order polynomial.
2. Diameter: The cross-section of the yarn is usually not a positive circle. The diameter can be approximated by the diameter of the major axis of the cross-section. The cross-section in the case of straightening should be perpendicular to the path of the center points, not identical to the cross-section on a slice along the coordinate’s axis. The normal plane perpendicular to the approximated path of the center points was used to obtain the cross-section of the yarn, and the radius of major axis of this cross-section was calculated using Equation (7). The process was repeated at a distance of 10 pixels on the axes between two adjacent steps. The average value was taken as the radius of the yarn.
3. Spacing: Adjacent yarns in the same direction are not strictly parallel. There is no spacing between two non-parallel lines. Because the angle between the yarns is relatively small, they could be approximated as two parallel lines within the ROI. The spacing between two adjacent yarns j and j+1 was calculated by the difference between the means of horizontal coordinates of the approximate paths:
  (10) $$d=\frac{1}{n}\sum_{i=0}^{n}x_{i}^{j+1}-\frac{1}{n}\sum_{i=0}^{n}x_{i}^{j}$$

## 3. Results and Discussion

### 3.1. Integration Interval

The ability of the orientation to characterize the features of the 3D volume image is closely related to the integration interval of the structure tensor. The natural inhomogeneity of the density of the sample is eliminated through the mean value over a large integration interval. However, the integration interval of pixels at the edge of the yarn contains pixels of other yarns and backgrounds. Integration intervals that are too large can instead induce new errors. 

**Figure 8:**
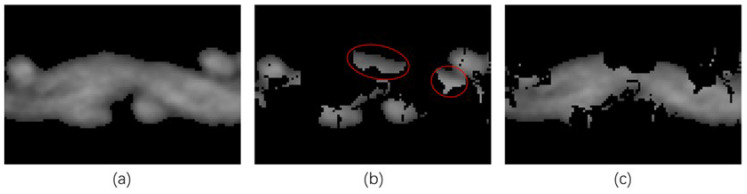
Results of segmentation when ω = 5: (**a**) The original grayscale image of the slice, where the wefts are perpendicular to the slicing plane and the warp is parallel to the slicing plane; (**b**) results of segmentation of the wefts; (**c**) results of segmentation of the warp. Wrongly segmented areas are indicated by red circles.

The size of the integration interval was obtained by comparing the accuracy of the orientation-based segmentation. The slice in Figure 8a was segmented by calculating the thresholds of the orientations obtained under different integration intervals by OTSU [40]. In the absence of ground truth, the segmentation effect was evaluated by the ratio of the area of the wefts (perpendicular to the slicing plane) and the warp (parallel to the slicing plane).

**Figure 9:**
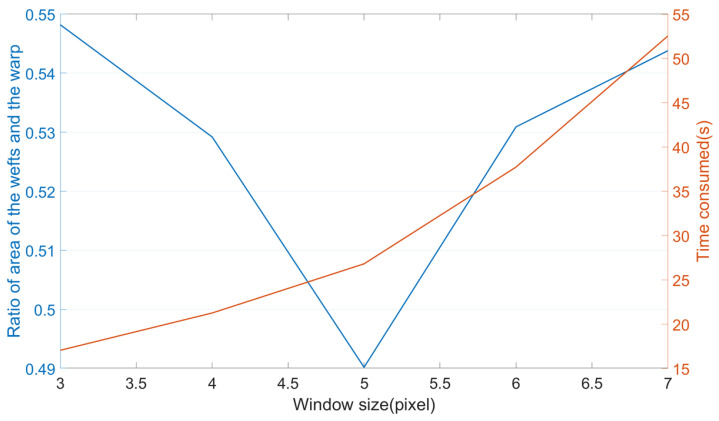
The ratios of the area of the wefts and warp (in blue) and the computation time (in orange) versus the integration interval.

Figure 9 demonstrates the effect of the integration interval on the segmentation. The ratio of the area of wefts to the warp was minimized when ω = 5. From the actual segmentation results shown in Figure 8b,c, it can be seen that the wefts were basically correctly classified. In contrast, the pixels in the red circle in Figure 8b that actually belonged to the warp were incorrectly labeled as wefts. The lower the ratio, the fewer pixels in the warp that were incorrectly segmented into the wefts, and the better the effect of characterization.

Meanwhile, the time consumed to calculate the structure tensor grew exponentially in relation to the integration interval. It ensured both an acceptable computation time and an excellent characterization effect when ω = 5.

### 3.2. Yarn Segmentation

The ground truth of segmentation on the slices of the CT-scanned images of wool fabrics were manually labeled on the grayscale images to validate the proposed method. All the cross-sections of yarns perpendicular to the slice plane were taken as positive samples, and those of yarns parallel to the slice plane were taken as negative samples, regardless of the background (i.e., zero-valued pixels) and the specific yarn they belonged to.

**Figure 10:**
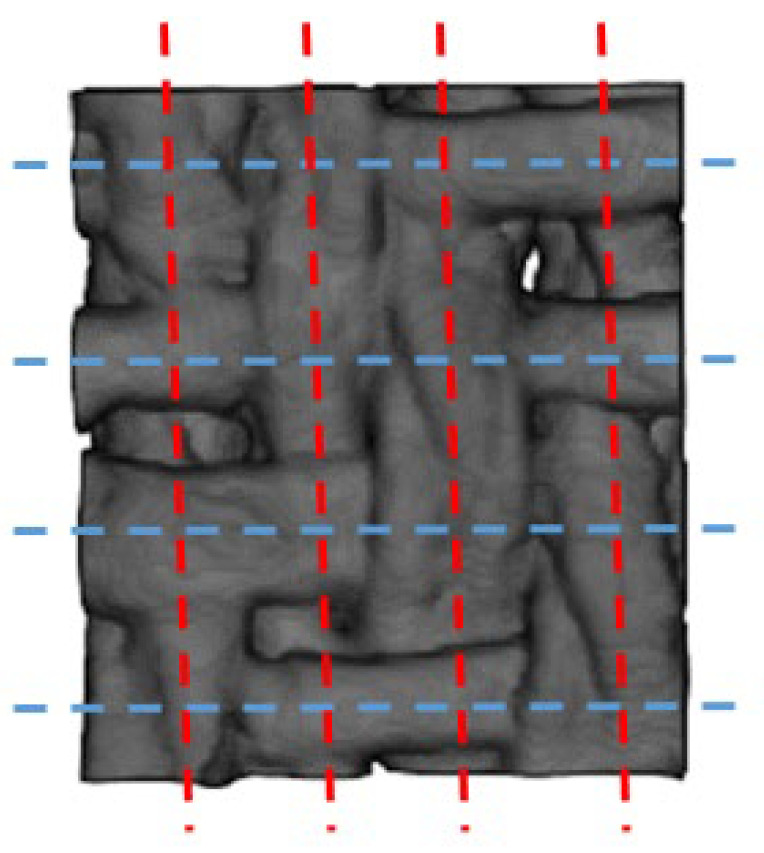
Location of the slices for evaluation in ROI No. 1 from the top view. The blue dashed lines indicate the four slices when the warps were taken as positive samples, and the red dashed lines indicate the four slices when the wefts were taken as positive samples.

The CT-scanned images of wool fabric are hard to manually label to establish the true values for each slice within the ROI. Ground truths were established only for the four slices with a local maximum of the area of wefts when the warps were used as positive samples, and vice versa. These eight slices were at the center of the weft or warp, parallel to their planes (Figure 10).

**Figure 11:**
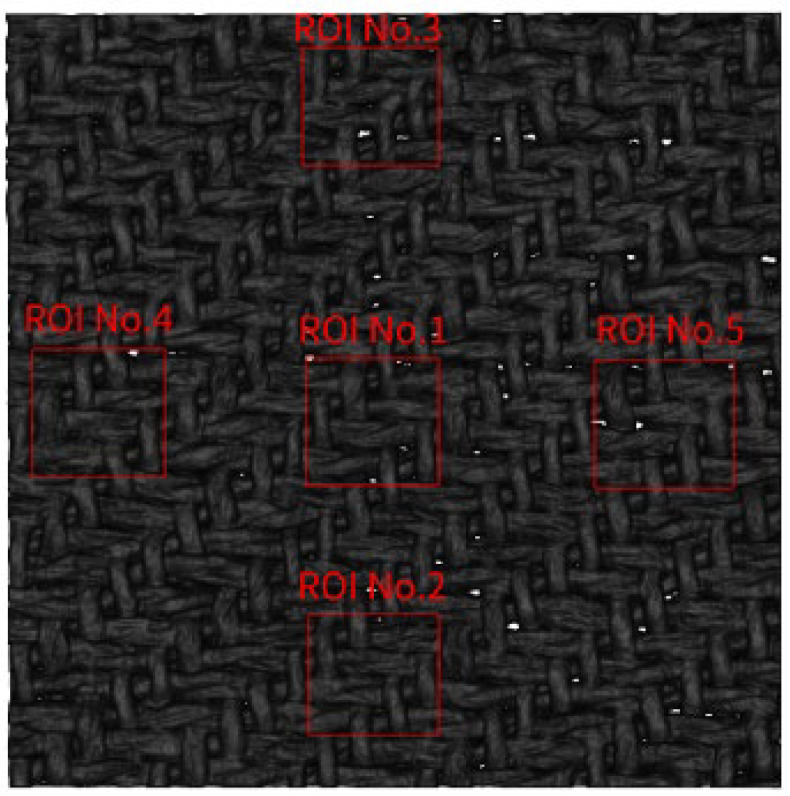
Location of the five ROIs in the sample.

**Figure 12:**
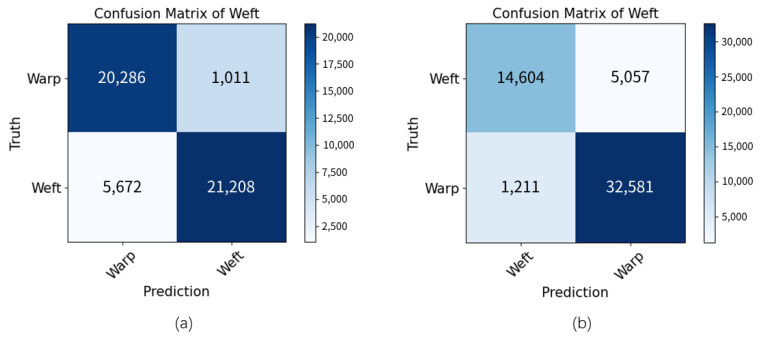
Confusion matrix: (**a**) when the warps were taken as positive samples, (**b**) when the wefts were taken as positive samples.

Five ROIs were selected for validation in the sample with the center, bottom side, top side, left side, and right side. Their positions are shown in Figure 11. Figure 12 illustrates the confusion matrix when the warps and the wefts were taken as positive samples. In semantic segmentation, the confusion matrix was pixel-based. 

PA and IOU have commonly been used as performance evaluation metrics. PA characterizes the proportion of the total number of pixels for which the prediction is correct. The elements on the diagonal of the confusion matrix represent the number of pixels correctly predicted in positive and negative samples. PA is the ratio of its sum to the sum of all elements. IOU represents the ratio of the intersection and concatenation of the predictions with the true values. In this task, IOU is calculated as the ratio of true positive samples to the sum of all samples except the true negative samples. Table 3 shows the PA and IOU when the positive samples were warps and wefts. The IOU exceeded 70% for both warps and wefts, and the PA exceeded 85% for both.

**Figure 13:**
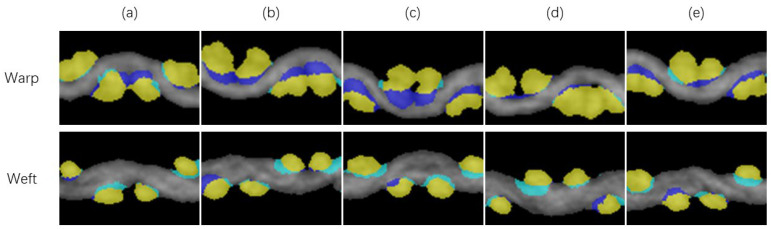
Comparison of the ground truth and predictions for yarn segmentation. The intersections are marked in yellow. Regions included only in the ground truth are marked in dark blue, and the regions included only in the predictions are marked in light blue. (**a**–**e**) mean 5 different slices.

Figure 13 shows the comparison between the predictions of the proposed method and the ground truth. In the visualization of the results, the periphery of the cross-section was well labeled. The main errors appeared in the part where the warps and wefts intersected. In the process of template selection, the orientation of zero-value pixels was modified to $\frac{\pi }{2}$, which was the theoretical mean orientation of the yarn parallel to the slicing plane. In practice, the radial direction of the warps and wefts and the plane where the fabric is located are not perfectly parallel to the three coordinate axes. There is a certain angle, making the mean orientation of the yarn parallel to the slicing plane different from the theoretical value. The template selection method will prefer to select the intersecting part when the set value of the orientation of zero-value pixels is larger. This value can be used as a penalty term to adjust the results of segmentation.

Table 3 also records the precision, recall and F1 score of the proposed method. These three metrics are usually used for classification problems rather than semantic segmentation. In the evaluation phase, it only considered whether the pixels belonged to the warps or the wefts (ignoring the few pixels that were discarded) and could be considered a pixel-level binary classification problem. In Table 4, the warp had a higher precision while the weft had a higher recall. This is related to the volume of the warps and wefts. The warps, as double-stranded yarns, had a larger cross-sectional area in the ground truth, while the wefts, as single-stranded yarns, had a smaller cross-sectional area. This meant that the warps would have fewer false positive pixels and the wefts would have fewer false negative pixels.

### 3.3. Feature Extraction

**Figure 14:**
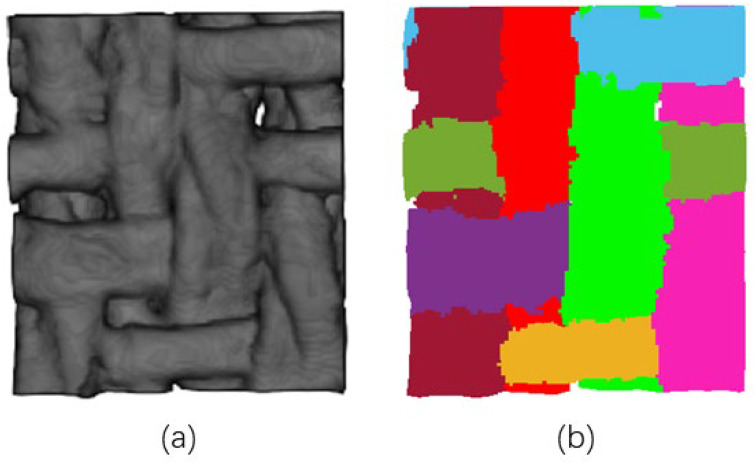
Top view of ROI No. 1: (**a**) preprocessed grayscale image; (**b**) results of yarn segmentation represented by different colors.

Figure 14 shows the 3D grayscale image after preprocessing and the results of yarn segmentation in ROI No. 1. Feature extraction was performed by the method defined in Section 2.5, and the results are recorded in Table 4 and Table 5.

The density of the warps and wefts are traditional characteristics of the textile industry. They refer to the number of yarns contained in a unit of length (10 cm was used in this study). In the microstructural characteristics, it is replaced by the yarns’ spacing. The densities in Table 4 and Table 5 were obtained by dividing the unit of length by the yarns’ spacing and were recorded to facilitate a comparison with the basic information of the sample. The relative error between the warp density of this ROI and of the sample was 1.0%, and it was 5.0% for the weft density. The finished wool fabric after treatment was an inhomogeneous object. 

A difference between the local and overall density of between 3% and 5% is acceptable in the textile industry. The warps of the sample were thicker, denser and more closely aligned with each other. They were less likely to be displaced than the wefts. The experimental results were consistent with this pattern.

Because of the inhomogeneity of the wool fabric, the diameter of the yarn could not be measured again by other methods as a control group. The standard deviation of the warps’ diameters was 0.011 mm and that of the wefts’ diameters was 0.023 mm. The trend was consistent with the fact that single-stranded yarns are more prone to deformation than double-stranded yarns. 

## 4. Conclusions

The purpose of this study was to automatically extract the microstructure of wool fabrics from the images scanned by micro-CT using image processing. The microstructure allows for more objective and precise control of the wool fabric’s properties during processing, replacing the traditional process design methods in the textile industry based on the experience of a team of engineers and repeated trial production.

The proposed two-step approach was developed for the unique characteristics of wool fabrics. The microstructure is estimated first by segmentation and then by extraction. First, the eigenvectors of the structure tensor are applied instead of grayscale values as features during segmentation to separate single yarns from the fabric in a template selection manner. Secondly, its parameters are obtained from the segmented yarns by means of central moments and polynomial fitting to reconstruct its geometric model. 

The method has considerable credibility and can provide both a reference for the wool fabric processing industry to control the products’ characteristics and to form a database for the subsequent development of deep learning. In the future, the relationship between the microstructure and the macroscopic properties, and the effects of processing on the microstructure will continue to be investigated, allowing the proposed method to further contribute to the development of intelligent production.

## Figures and Tables

**Table 1:** Basic information of the sample.

	Weight (g/m)	Width (cm)	Warp Density (yarns/10 cm)	Weft Density (yarns/10 cm)	Warp Count	Weft Count
Property	260	145	300	260	54S/2	54S/1

**Table 2:** Operating parameters of the micro-CT scanner.

Parameters	Value
Source voltage (kV)	50
Source current (μA)	200
Rotation step (deg)	0.2
Frame averaging	4
Image pixel size (μm × μm)	15 × 15

**Table 3:** Experimental results of the proposed method of yarn segmentation.

	IOU (%)	PA (%)	Precision (%)	Recall (%)	F1 Score (%)
Warp	75.46	86.13	95.25	78.15	85.86
Weft	70.34	88.27	74.28	92.34	82.33

**Table 4:** Microstructural features of the warps in the five ROIs.

	Diameter (mm)	Spacing (mm)	Local Density (yarns/10 cm)	Global Density (yarns/10 cm)
ROI No. 1	0.38	0.33	303	300
ROI No. 2	0.40	0.33	303	
ROI No. 3	0.38	0.33	303	
ROI No. 4	0.37	0.33	303	
ROI No. 5	0.38	0.33	303	

**Table 5:** Microstructural features of the wefts in the five ROIs.

	Diameter (mm)	Spacing (mm)	Local Density (yarns/10 cm)	Global Density (yarns/10 cm)
ROI No. 1	0.30	0.41	247	260
ROI No. 2	0.31	0.39	256	
ROI No. 3	0.29	0.39	256	
ROI No. 4	0.35	0.39	256	
ROI No. 5	0.34	0.38	267	

**Figure A1:**
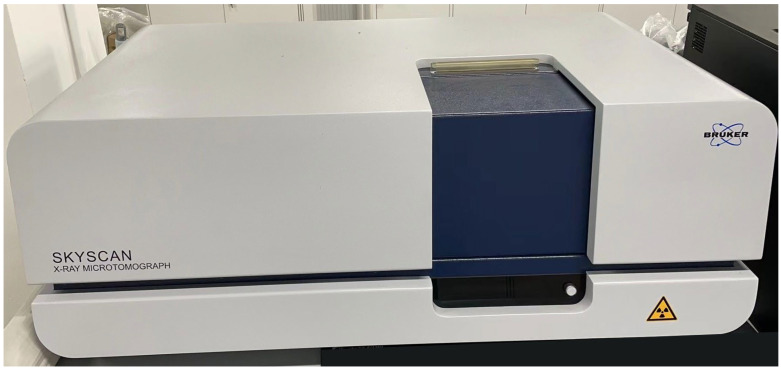
SkyScan-1275 Micro-CT scanner.

**Figure A2:**
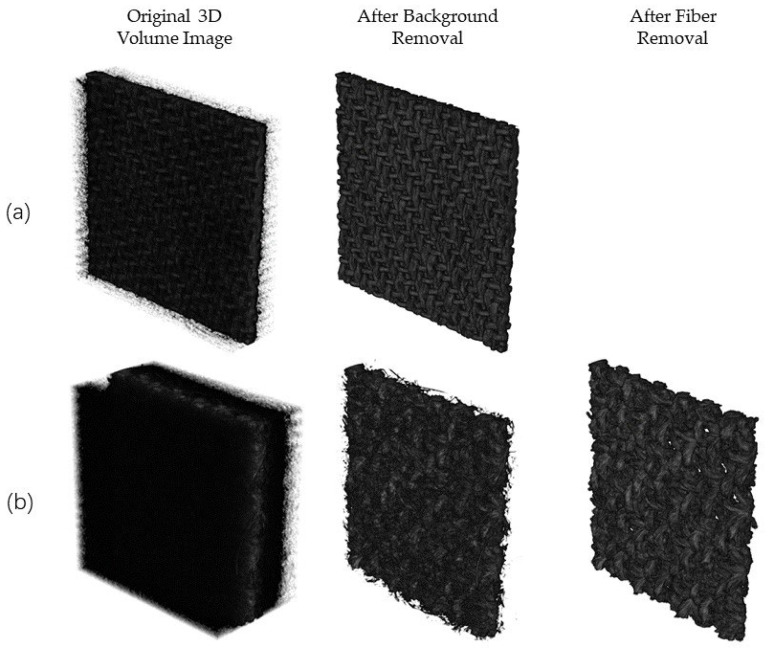
The effect of the two main steps of preprocessing. (**a**) Sample A is the one shown in Section 2.1. (**b**) Sample B is another example used to show the effect of fiber removal.

## Data Availability

The data are not publicly available due to the company’s requirements.

## Appendix A

The instrument used in the experiment was aSkyScan-1275 Micro-CT scanner from Bruker, Billerica, MA, United States. The appearance of the device is shown in Figure A1.

## Appendix B

Figure A2 shows the effect of the two main steps of the preprocessing described in Section 2.1 on the 3D volume images of the samples: background removal based on OTSU and fiber removal based on morphological processing. The sample shown in Section 2.1 had no fibers on its surface. No morphological treatment was applied to it. The second row of Figure A2 demonstrates the effect of fiber removal with another sample possessing abundant fibers.
